# SARS-CoV-2 surface contamination in metro-Atlanta grocery stores

**DOI:** 10.1371/journal.pone.0291747

**Published:** 2023-09-19

**Authors:** Travis W. Brown, Geun W. Park, Beth Wittry, Leslie Barclay, Margaret Person, Boris Relja, Scott Daly, Preeti Chhabra, Erin Kincaid, Jona Johnson, Ausaf Ahmad, Owen Herzegh, Jan Vinjé, Jennifer Murphy

**Affiliations:** 1 Division of Foodborne, Waterborne, and Environmental Diseases, National Center for Emerging Zoonotic and Infectious Diseases, Centers for Disease Control and Prevention, Atlanta, Georgia, United States of America; 2 Division of Viral Diseases, National Center for Immunological and Respiratory Diseases, Centers for Disease Control and Prevention, Atlanta, Georgia, United States of America; 3 Division of Environmental Health Science and Practice, National Center for Environmental Health, Centers for Disease Control and Prevention, Atlanta, Georgia, United States of America; 4 Division of Scientific Resources, National Center for Emerging Zoonotic and Infectious Diseases, Centers for Disease Control and Prevention, Atlanta, Georgia, United States of America; University of British Columbia, CANADA

## Abstract

While the COVID-19 pandemic has had a detrimental impact on many businesses worldwide, essential businesses, such as grocery stores, continued to operate despite potential disease transmission. Although the principal mode by which people are infected with SARS-CoV-2, the virus that causes COVID-19, is through exposure to respiratory droplets and very small particles carrying infectious virus, contaminated surfaces might play a role in transmission. We collected swab samples from frequently touched surfaces, including grocery carts, touchscreen monitors, credit card keypads, pharmacy counters, self-service food utensils, and refrigerator and freezer handles, in two metro-Atlanta grocery stores over the course of two sampling events in March 2021. Of the 260 swab samples collected, 6 (2.3%) samples were positive for SARS-CoV-2 RNA by reverse transcriptase quantitative polymerase chain reaction. Positive samples were collected from pharmacy (12.0% [3/25] samples), refrigerator/freezer aisles (2.5% [1/39] samples), and self-service food court (5.0% [2/40] samples) areas. Table/counter edge and underside surfaces represented 33% (2/6) of positive samples. These data suggest that risk of exposure to SARS-CoV-2 from frequently touched surfaces in grocery store settings is likely low; however, more frequent cleaning of surfaces in pharmacy and self-service food courts might be warranted.

## Introduction

The principal mode by which people are infected with SARS-CoV-2, the virus that causes COVID-19, is through exposure to respiratory fluids containing infectious virus [1]. Exposure to infectious respiratory fluids occur through the following pathways: (1) inhalation of very fine respiratory droplets and aerosol particles, (2) deposition of respiratory droplets and particles on exposed mucous membranes, and (3) touching mucous membranes with hands that have been soiled either directly by virus-containing respiratory fluids or indirectly by touching surfaces with virus on them. Respiratory fluids are excreted during any form of exhalation, including breathing, speaking, singing, coughing and sneezing [2–4], and consequently, are dispersed into the environment where they can be deposited onto surfaces [5]. The risk of surface-mediated transmission is dependent on several factors, including rate of COVID-19 prevalence within a community, survivability of virus on the surface and the influencing environmental factors (e.g., temperature, humidity, sunlight, etc.), the time between when a surface becomes contaminated and is touched, and the efficiency in which virus transfers from the surface to hands and hands to mucous membranes. When combining all these factors, the risk of surface transmission relative to other modes of transmission is thought to be low [6]. However, there might be risk of surface transmission in some settings where COVID-19 prevalence is high and prevention measures (e.g., surface cleaning and hand hygiene) are difficult to maintain.

Essential businesses, such as grocery stores, remained open during the COVID-19 pandemic despite widespread community transmission. Grocery stores present a setting where there might be an elevated risk of surface-mediated transmission due to the presence of high numbers of potentially infected customers within relatively short periods of time. Furthermore, grocery stores contain many frequently touched surfaces, such as grocery cart handles, credit card keypads, and door handles, where viruses might be present if surfaces are not routinely cleaned. Current guidance recommends regularcleaning of frequently touched surfaces and hand hygiene measures (e.g., handwashing with soap and water or using hand sanitizers containing at least 60% alcohol if soap and water are not readily available) as protective measures against infection [7, 8].

SARS-CoV-2 surface contamination has been widely documented in healthcare settings, but less so in community spaces, such as a grocery stores [9–11]. A systematic review of SARS-CoV-2 surface contamination published in December 2021 found that 10.2% of surfaces sampled tested positive in non-hospital settings, including outdoor and indoor community environments in urban and rural settings (e.g., public transportation, essential businesses, education centers) [12]. Three studies that evaluated SARS-CoV-2 RNA surface contamination in grocery stores in the United States (April-June 2020), Canada (October-November 2020) and Italy (April-May 2021) reported surface positivity rates of 11.1%, <0.1%, and 4.3%, respectively [13–15]. A corresponding quantitative microbial risk assessment (QMRA) in the U.S. study concluded that risk of infection from exposure to a contaminated surface to be low (<5 in 10,000).

While current evidence suggests that contaminated surfaces in grocery stores present a low risk of infection, a robust examination of frequently touched surfaces in grocery store settings, especially those that might be missed during routine cleaning, provides important information that can inform public health disease prevention measures. Furthermore, specific areas of the store, such as the pharmacy, might present higher frequency and levels of surface contamination and warrant focused examination. This study evaluated SARS-CoV-2 surface contamination in two metro-Atlanta grocery stores in March 2021. The objectives of this study were to (1) determine the extent of SARS-CoV-2 surface contamination across the two grocery stores, and (2) document the areas of the store and types of surfaces that are prone to surface contamination to better inform cleaning practices.

## Materials & methods

### Grocery store selection

Two grocery stores (Store A and Store B) of a major grocery store chain in the metro-Atlanta area were selected in collaboration with regional store management. Store selection was based on a convenience sampling approach by regional anagement. Preliminary store visits were conducted to collect observations of the type of frequently touched surfaces and to develop a sampling framework. Two sampling events at each store location were scheduled for March 6 and 27, 2021. This activity was reviewed by CDC and was conducted consistent with applicable federal law and CDC policy.

### Sample collection

The surface swabbing method used was based on a method developed for environmental sampling for norovirus and has recently been applied for SARS-CoV-2 [16–19]. Prior to sample collection, dry macrofoam swabs (Sanigen Co.; South Korea) with a head size of 19 mm x 26 mm were pre-moistened with 7.5 ml of sterile phosphate buffered saline (PBS) added directly into the swab tubes. PBS alone served as the swab eluent, as detergents have been shown to disrupt SARS-CoV-2 viability [20, 21].

Based on the estimated time required to collect surface swab samples (2–3 hours), the number of available field staff, and time and staff needed for laboratory processing, a sample size of 250 surface swabs across two stores and two sampling events in March 2021 was targeted. Sampling plans were developed for each store location to target collection of 60–70 samples from frequently touched surfaces in the following customer and employee areas: pharmacy, refrigerator and freezer aisles, customer check-out, self-service food court, customer bathrooms, facility operations, preparatory kitchens, and employee break rooms (Table 1). Sampling dates and times were scheduled during or shortly following periods of high customer utilization, as reported by regional store management. To our knowledge, date and time of sampling was not disclosed with local store management or employees. Researchers instructed managers to encourage store employees to continue store-mandated standard cleaning and disinfection practices.

**Table 1:** Surface areas swabbed in two grocery stores of a major grocery store chain in the metro-Atlanta area.

Grocery Store Area	Surface Type Sampled
Pharmacy	Counters (underside/edge and top), touchscreens, POS machines, chairs
Refrigerator/Freezer Aisles	Refrigerator/freezer door handles
Customer Checkout	Touchscreens, POS^a^ machines, conveyor belt dividers
Self-Service Food Court	Dining tables (underside/edge and top), chairs, tongs, bins
Customer Bathrooms	Toilet levers, door handles, sink knobs
Facility Operations	Telephones, computer keyboards, loading dock equipment
Prep Kitchens	Ice machine lids and scoops, refrigerator/freezer door handles
Employee Break Rooms	Dining tables, chairs, microwaves,
Miscellaneous	Grocery carts, baskets, ATM machines

^**a**^ POS: point of sale

CDC subject matter experts trained CDC field staff to collect samples from pre-identified frequently touched surfaces in both customer and employee areas and to sample any additional surfaces that were actively being touched at the time of the sampling or those that appeared to have been missed during routine cleaning. At each sampling site, pertinent information (e.g., date and time of collection, area of store, detailed description of surface and location) was documented. A measuring tape was used to estimate surface area in cm^2^ of swabbed surface to allow for backcalculation of PCR data to gene copies per cm^2^. At each site, a sample collector donned a new pair of nitrile gloves, removed a pre-moistened swab from tube, and gently pressed the swab against the sides of tube to remove excess eluent. The entire surface area was sampled by swabbing one time each in horizontal, vertical, and diagonal directions. In some instances, composite samples of similar adjacent surfaces were collected to utilize the maximum surface area capacity of each swab (645 cm^2^) (e.g., 1 swab for 4 adjacent freezer door handles at ~160 cm^2^ each). The swab was then immediately transferred back into the tube and placed in a cooler with ice packs. Following sampling, swabbed surfaces were cleaned with a disinfecting wipe (Lysol; Slough, England, UK) to remove residual PBS and for customer perception purposes. Swab samples were transported back to CDC Atlanta the same day and stored at -20°C until analysis.

### Surface swab elution and concentration

Surface swabs collected from the first and second sampling events were processed in an enhanced BSL-2 environment (BSL-2 with BSL-3 safety and containment practices) within 32–34 days and 11–16 days of collection, respectively. After thawing at room temperature, each swab tube was vortexed for 10 seconds. Next, the swab tube was carefully opened and the swab was pressed gently against the sides of the tube to extract eluant. Eluant was transferred to a 15 ml conical tube and centrifuged for 10 minutes at 4,000 × *g* to pellet particulate matter that could clog centrifugal filters during concentration. The eluant supernatant was transferred to a new 15 ml conical tube and approximately 4 ml was then transferred to a 30,000 kDa Amicon Ultra-4 filtration device (Millipore Sigma; Burlington, MA). The Amicon filtration device was centrifuged at 4,000 × *g* for 10 minutes to concentrate eluant to the graduated 250 μl mark. If eluant was concentrated to a volume greater than 250 μl, sample was centrifuged an additional 5–10 minutes to achieve 250 μl; if eluant was concentrated to a volume less than 250 μl, additional sterile PBS was added to the filter to achieve desired volume. Concentrated eluant was then resuspended within the filtration device using a micropipette and transferred to a sterile 1.7 ml microcentrifuge tube. The eluant remaining in the 15 ml conical tube (~1 ml) was stored in 0.1% fetal bovine serum (FBS) and transferred to -80°C for subsequent SARS-CoV-2 infectivity assay, if the RT-qPCR signal indicated sufficient RNA concentration to attempt culture (cycle threshold [Ct] of ~30 or less) [22, 23].

### Viral RNA extraction and reverse transcription–Realtime polymerase chain reaction

Immediately following sample concentration, viral RNA was extracted using an automated QIAcube HT nucleic acid extractor using the QIAamp 96 Virus QIAcube HT Kit (QIAGEN; Germantown, MD), according to manufacturer’s recommendations. Briefly, concentrate volumes of 200 μl were added to individual wells of a 96 deep-well plate containing 160 μl of ACL buffer, 20 μl proteinase K, and 5 μl of an A549 cell suspension at 10^6^ cells/ml as a Human Specimen Control (HSC) internal control to monitor extraction integrity [18]. On each plate, a seasonal influenza positive control (SIPC) containing both influenza A and B was included as a positive control and sterile PBS was included as an extraction blank.

Extracted RNA was tested for SARS-CoV-2 in duplicate using the validated CDC influenza SARS CoV-2 (Flu SC2) multiplex assay on the Applied Biosystems 7500 Fast Dx Real-Time PCR instrument (Thermo Fisher Scientific; Waltham, MA) as described previously [24]. While the Flu SC2 assay was used in this study, the influenza targets were not a goal of the study and results are not reported. The Flu SC2 assay was the approved and validated assay for SARS-CoV-2 detection by the testing laboratory at the time of analysis. In each RT-qPCR run, a 10-fold serial dilution (1–10^6^ copies/μl) of SARS CoV-2 RNA transcript were tested in triplicate to generate a standard curve. The average slope and deviation of the standard curves were -3.2073 ± 0.0545 (R^2^ > 0.997), with a reaction efficiency of 105%. The concentration of SARS CoV-2 was calculated by converting Ct values of the standard curve to RNA copies. A sample was considered positive if at least 1 of the 2 replicates had a Ct below 40. A combined influenza and SARS-CoV-2 positive control (SIPC + SC2 RNA), extraction blanks, and no template controls (NTCs) were included on each plate.

## Results

Of the 260 surface samples collected from both stores on two separate sampling events, the overall sampling positivity was 2.3% (6/260). Sampling positivity by area of store was as follows:12.0% (3/25 samples) in pharmacy areas, 2.5% (1/39 samples) in refrigerator/freezer aisles, and 5.0% (2/40 samples) in the self-service food courts. No (0/130) samples from Store A tested positive for SARS-CoV-2 by RT-qPCR while 4.6% (6/130) samples from Store B tested positive (3 samples were positive at each sampling event) (Tables 2 and 3). The three Store B areas with positive samples included the pharmacy (25.0% [3/12] samples), refrigerator/freezer aisles (4.3% [1/23] samples), and self-service food court (9.5% [2/21] samples). One half of positive samples (3/6 samples) were collected in the pharmacy area of Store B. Two positive samples correspond to table/counter underside and edge surfaces (1 in the pharmacy and 1 in the self-service food court); in each case, RNA was not detected in samples from the top of the corresponding table or counter. The remaining positive samples were a touchscreen monitor for clinic registration and a chair armrest in the pharmacy, a bread-bin in the self-service food court, and a refrigerator door handle in the refrigerator/freezer aisle (Table 3).

**Table 2:** Number (%) of surface swabs^a^ collected from grocery stores A and B positive for SARS-CoV-2 RNA by RT-qPCR, March 2021.

	Number (%) of Surface Swabs Positive for SARS-CoV-2 RNA		
Grocery Store Area	Store A	Store B	Store A + B
Pharmacy	0/13 (0.0%)	3/12 (25.0%)	3/25 (12.0%)
Refrigerator/Freezer Aisles	0/16 (0.0%)	1/23 (4.3%)	1/39 (2.5%)
Customer Checkout	0/16 (0.0%)	0/13 (0.0%)	0/29 (0.0%)
Self Service Food Court	0/19 (0.0%)	2/21 (9.5%)	2/40 (5.0%)
Customer Bathrooms	0/15 (0.0%)	0/10 (0.0%)	0/25 (0.0%)
Facility Operations	0/15 (0.0%)	0/9 (0.0%)	0/24 (0.0%)
Prep Kitchens	0/11 (0.0%)	0/16 (0.0%)	0/27 (0.0%)
Employee Break Rooms	0/17 (0.0%)	0/15 (0.0%)	0/32 (0.0%)
Miscellaneous	0/8 (0.0%)	0/11 (0.0%)	0/19 (0.0%)
**All Areas**	**0/130 (0.0%)**	**6/130 (4.6%)**	**6/260 (2.3%)**

^a^In some instances, a single surface swab was a composite of numerous similar and adjacent surfaces to maximize surface area capacity of swab (645 cm^2^)

**Table 3:** Location and description of samples positive for SARS-CoV-2 RNA by RT-qPCR.

Store ID	Sampling Event^a^	Grocery Store Area	Surface Type Sampled	N gene gc/cm^b^
Store B	1	Pharmacy	Chair armrest in customer waiting area	0.2
Store B	1	Pharmacy	Prescription pick-up counter underside and edge	0.3
Store B	1	Refrigerator/Freezer Aisles	Refrigerator door handle	1.3
Store B	2	Pharmacy	Touchscreen monitor for walk-in medical clinic registration	1.8
Store B	2	Self Service Food Court	Dining table underside and edge	1.0
Store B	2	Self Service Food Court	Lid and tongs of bread container	2.1

^a^Sampling event 1: March 6, 2021; Sampling event 2: March 27, 2021

^b^ N gene gc = SARS-CoV-2 nucelocapsid genome copies

RNA concentrations in positive samples ranged from 0.2–2.1 genomic copies/cm^2^. SARS-CoV-2 concentrations recovered from surfaces were too low (Ct >30) to attempt SARS-CoV-2 culture, therefore presence of infectious virus in samples could not be determined. On each qPCR run, all positive controls tested positive and all NTCs and extraction blanks tested negative. The RNase P target from the HSC internal control tested positive in all samples, indicating sufficient RNA extraction in samples.

## Discussion

While SARS-CoV-2 transmission from contaminated surfaces is thought to be low, contaminated surfaces in grocery stores might present an increased risk of surface transmission. An evaluation of SARS-CoV-2 RNA contamination on surfaces was conducted in two metro-Atlanta grocery stores in March 2021. Store B was the only store to have detectable RNA on surfaces (n = 6); concentrations recovered were too low to attempt culture and determine presence of infectious virus. The low overall positivity rate (2.3%) and low concentrations of virus (0.2–2.1 gc/cm^2^) recovered from frequently touched surfaces suggest that risk of SARS-CoV-2 exposure from contaminated surfaces in grocery stores is likely low.

The grocery stores that were included in this study were part of a larger retail grocery chain and these stores might have more resources to institutionalize cleaning and disinfection practices. The stores’ surface cleaning and disinfection practices might have played a role in the low rates of viral contamination on surfaces [25]. Additionally, the relative level of COVID-19 community spread might explain low rates of surface contamination. At the first sampling event, Store A and Store B county COVID-19 case rates from previous two weeks were 233 and 296 cases per 100,000, respectively, according to Georgia Department of Public Health data [26]. For the second sampling event, county COVID-19 case rates from previous two weeks were 132 and 211 cases per 100,000. For reference, national-level case rates were in a period of decline from a peak in cases in January 2021, according to CDC COVID Data Tracker [27]. This trend is also observed at the state-level; case rates during the time of sampling were lower compared to Georgia two-week case rates documented in January 2021 (840–1,142 cases per 100,000). While case rates in the Store B county are slightly higher than the Store A county, case rates alone likely do not explain the discrepancy of detectable RNA between stores. Other factors may include community behaviors (e.g., masking), store occupancy rates, and differences in store cleaning practices.

While surface positivity was low overall, 3 of 6 (50%) samples that tested positive for SARS-CoV-2 RNA were collected from the pharmacy area. This was also the only area with positive samples during both sampling events, suggesting that contamination is likely a persistent problem in this area. A potential explanation might be that (1) the pharmacy is visited by those that might be unwell and experiencing COVID-19 symptoms and (2) this is an area of the store that can experience high occupancy and relatively long service wait-times in a potentially confined area. Together, these factors might explain more frequent contamination in the pharmacy, as compared to other areas of the store. Increased frequency of cleaning or more thorough cleaning might be necessary for pharmacy areas to reduce exposures of customers and store employees to contaminated surfaces, especially considering the pharmacy might service individuals more susceptible to severe COVID-19 outcomes. This same approach might also be necessary for the self-service food court area, from which 2 of 6 (33%) of positive samples were collected. This area of the store likely experiences a higher volume of unmasked customers than other areas due to the presence of a dining area where food is actively consumed. Interestingly, surface types that were most frequently positive were collected from table/counter underside and edge surfaces in the pharmacy and self-service food court. These surfaces are frequently touched but might be missed during routine environmental cleaning.

This study contributes to the growing body of literature reporting detectable, but low concentration SARS-CoV-2 surface contamination in community settings [12, 28, 29]. Specifically in grocery store settings, studies in the United States (Massachusetts), Canada, and Italy, reported surface positivity rates of 11.1%, <0.1%, and 4.3%, compared to the 2.3% reported in this study [13–15]. Different positivity rates might be attributed to the level of community spread of SARS-CoV-2 at the time of sampling, the COVID-19 prevention measures implemented (e.g., frequency of cleaning), and behaviors (e.g., masking). The Massachusetts study also reported concentrations of SARS-CoV-2 RNA recovered from surfaces. While they reported higher concentrations (2.5–11.6 gc/cm^2^) than reported here (0.1–2.1 gc/cm^2^), the concentrations reported in both studies suggest relatively low exposure levels from contaminated surfaces.

This study has several limitations. First, only two stores were each sampled twice in March 2021, and more data, including observational and behavioral data, would be needed during different phases of the pandemic to confirm the low surface exposure risk in grocery stores. Additionally, the conclusions of this study are limited to the study locations and cannot be extrapolated to other grocery stores or other retail businesses. Second, stores might have cleaned surfaces before sampling events; therefore, results might present at underestimate of SARS-CoV-2 surface contamination. Third, due to biosafety restrictions in the laboratory, we were unable to immediately process samples after collection. Therefore, samples underwent a freeze-thaw step that potentially diminished chances of detection in samples with very low RNA concentrations as freeze-thaw cycles likely have an impact on the SARS-CoV-2 recovery from surface swabs. Additionally, samples were mistakenly stored at -20°C and may have influenced virus decay; the optimal temperature for storing virus samples is -80°C. Fourth, we were unable to determine whether the positive samples were infectious due to low levels of SARS-CoV-2 RNA recovered. Inclusion of infectivity data would assist in characterizing risk of exposure; RNA detected might have been from non-infectious virus, perhaps inactivated from previous cleaning or disinfection of the surface or from natural decay. And lastly, the swabbing method used in this study needs further validation to determine recovery efficiency of SARS-CoV-2 from surfaces. It is unclear if the low concentrations of virus reported in this study were from actual low level surface contamination or poor method performance.

Grocery stores, pharmacies, and other retail businesses that remain open regardless of the extent of local COVID-19 spread are settings where surface-mediated transmission might be a concern. While a formal exposure assessment was not conducted in this study, our data suggest that exposure to SARS-CoV-2 from frequently touched surfaces in grocery store settings is likely low. However, surface mediated transmission is still possible and prevention measures implemented by both store managers and employees (e.g., regular cleaning of surfaces) and customers (e.g., hand hygiene) remain important prevention measures. Based on our limited sampling events, additional studies examing RNA and infectious virus surface contamination with corresponding behavioral and observational datain grocery stores and other retail business settings in additional geographic locations, and particularly in pharmacy and self-service food areas, might be warranted.

## Supporting information

S1 Data (XLSX)
